# Faster-setting calcium silicate cements–composite bonding: effect of adhesive application mode

**DOI:** 10.1186/s12903-026-08465-6

**Published:** 2026-04-28

**Authors:** Zeliha Gonca Bek Kürklü, Ezgi Sonkaya

**Affiliations:** Faculty of Dentistry, Department of Restorative Dentistry, Cukurova University, Sarıcam, Adana, Türkiye

**Keywords:** Biodentine, Failure mode, Faster-setting calcium silicate cements, Micro-shear bond strength, NeoMTA 2, White MTA Angelus

## Abstract

**Background:**

Calcium silicate cements are widely used as pulp-capping agents, liners/bases, and repair materials in vital pulp therapy and endodontic procedures. Owing to their biocompatibility and sealing ability, they are typically placed beneath definitive restorations. This study aimed to compare the micro-shear bond strength of three faster-setting (short initial setting time) calcium silicate cements to a resin composite and to determine whether bond failure is predominantly adhesive at the interface or cohesive within the cement.

**Methods:**

Sixty cylindrical specimens were prepared of three calcium silicate cements (CSCs) products White MTA Angelus, NeoMTA 2, and Biodentine. For each cement, two adhesive application protocols were tested (passive, P, and active, A: *n* = 10 per subgroup). The adhesive was applied to the calcium silicate cements and layer was light-cured, and a flowable resin composite was placed on the cement surface and also light-cured. After incubation at 37 °C for 24 h, the interfaces were subjected to micro-shear bond strength testing at a crosshead speed of 0.5 mm/min. Data were analyzed using one-way analysis of variance followed by Tukey’s post hoc test (α = 0.05). Failure modes were examined under a stereomicroscope.

**Results:**

Significant differences in micro-shear bond strength (MPa) were observed among cement types (*p* < 0.001). Biodentine yielded higher values (BA: 4.5 ± 2.8; BP: 6.1 ± 4.1) than White MTA Angelus (AA: 1.5 ± 0.4; AP: 2.1 ± 0.4) and NeoMTA 2 (NA: 1.8 ± 0.7; NP: 1.0 ± 0.5). No significant difference was found between passive and active adhesive application within the same cement (*p* > 0.05). Cohesive failure predominated across all groups (> 70% of failures), and NeoMTA 2 exhibited 100% cohesive failure.

**Conclusions:**

The micro-shear bond strength (µSBS) between faster-setting calcium silicate cements and a flowable resin composite appeared to be limited primarily by the cement’s cohesive strength rather than at the adhesive interface. Biodentine showed higher 24-hour µSBS values compared with the other CSCs when a definitive restoration is placed in a single visit. These findings highlight the importance of considering the early µSBS and the cement’s cohesive strength when selecting a base for immediate restorative placement.

## Background

Vital pulp therapy is important in modern conservative dentistry, aiming to maintain the vitality and function of the dental pulp while benefiting from the tooth’s natural defense and repair capacity. Calcium silicate–based cements (CSCs) dental products, often denoted as mineral trioxide aggregate (MTA), are often used for vital pulp therapy, perforation repair, apexification, and retrograde filling due to their high biocompatibility, antibacterial pH, mineralization (bioactivity), and lack of shrinkage [1].

However, some drawbacks of CSCs have been prolonged setting times, reported in the literature to extend up to 228 min [2]. Originally, a definitive restoration was sometimes delayed for orthograde vital pulp therapy (VPT), with a second appointment preferred to allow further strengthening of the cement [3, 4]. Newer CSCs having setting times of only 12–15 min (e.g., Biodentine, NeoMTA 2, White MTA Angelus) have been developed. It is recommended to cover the CSC surface with a glass ionomer, a light-curable resin-modified glass ionomer cement or a flowable composite as an intermediate layer, the latter followed by placement of the permanent restoration in the same session. This approach allows the CSC to complete its setting process safely beneath the restoration [5].

In this context, the CSC–resin interface is a practical consideration for one-visit restorative workflows, rather than a prerequisite for the biological success of vital pulp therapy. Although true chemical adhesion between CSCs and resin materials is not necessarily expected, any interfacial retention is likely to depend on limited micromechanical interlocking and adaptation to the surface irregularities of the cement. According to the manufacturer’s documentation for Biodentine [6], the use of an adhesive-based definitive resin restoration is explicitly described; the corresponding adhesive application step is not detailed to the same extent in the manufacturers’ documentation for the other CSCs. However, since dental adhesives are designed for enamel and dentin, the predictability of bonding to calcium silicate cement surfaces, particularly at early time points, such as 15 min after placement, should be experimentally validated. The polymerization shrinkage stress of flowable composites and resin-modified glass ionomer cements has been reported to range from 0.33 to 10.45 MPa [7–9]. Therefore, reported polymerization-shrinkage stresses in overlying restorative materials provide a rationale for evaluating early CSC–restorative bonding performance and failure modes.

The interfacial bond strength between CSCs and resin has been widely investigated using the shear bond strength (SBS) test. Previous studies have evaluated the influence of different adhesive strategies [5, 10–15], compared immediate and delayed bonding protocols [5, 15–17], and examined the adhesion of CSCs to various restorative materials [16–21]. For example, Palma et al. [22] reported mean SBS values of 1.3 ± 1.6 MPa for ProRoot MTA–flowable composite and 4.4 ± 2.5 MPa for Biodentine–flowable composite when bonding was performed 12 min after material placement. Çeliksöz et al. [16] reported Biodentine–composite µSBS values when bonding was completed at 3, 12 and 24 h, and tested at 24 h after bonding— clarify and rewrite please….of 1.8 ± 0.2 MPa at 3 min, increasing to 6.0 ± 0.6 MPa at 12 min and 6.4 ± 0.4 MPa at 24 h. Most SBS studies have reported predominantly cohesive or mixed fracture patterns during testing [5, 11, 12, 14, 17, 18, 21, 23].

This study aimed to evaluate the micro-shear bond strength (µSBS) between three faster-setting calcium silicate-based cements (MTA Angelus, NeoMTA 2, and Biodentine) and a flowable resin composite, and to compare passive and active adhesive application modes, because active application may promote better adhesive adaptation to the cement surface, while also posing a risk of disrupting the mechanically immature substrate. Failure patterns were analyzed to determine whether failures were primarily due to interfacial adhesion or to the intrinsic mechanical strength of the cements.

The null hypotheses were: (1) no significant difference in bond strength among the three calcium silicate cements; (2) no significant difference between passive and active application of the adhesive; and (3) the dominant failure mode would neither differ among cements nor be limited by internal cohesive strength.

## Methods

### Specimen preparation

Sixty acrylic blocks were fabricated using Teflon molds, each containing a central cylindrical cavity measuring 2 mm in depth and 4 mm in diameter. The CSC products were: MTA Angelus (Angelus, Brazil), NeoMTA 2 (Avalon Biomed, USA), and Biodentine (Septodont, France) Table 1 has information on their components. Each was mixed and handled according to the manufacturers’ instructions and placed into the prepared cavities using a spatula. To standardize the bonding surface, a glass slide was placed over the molds during placement and initial setting to obtain a flat surface.

**Table 1:** Chemical components and MIXING procedure of calcium silicate cements*

Materials	Manufacturer	Components	MIXING	Initial Setting Time (min.)
White MTA Angelus	Angelus, Brazil	Powder: Tricalcium silicate, Dicalcium silicate, Tricalcium aluminate, Tetracalcium aluminoferrite, Calcium oxide, Calcium tungstate Liquid: Distilled Water	Mix 1 sachet powder with one drop of liquid	10 (final set 15)
NeoMTA 2	NuSmile, USA	Powder: Tricalcium silicate, Tantalite / Tantalum pentoxide, Dicalcium silicate, Calcium sulfate, Tricalcium aluminate Liquid: Water-based gel	Mix 1 small scoop of powder with one drop of gel	15
Biodentine	Septodont, France	Powder: Tricalcium silicate, Calcium carbonate, Zirconium oxide Liquid: Water, Calcium chloride, Modified polycarboxylate	Add 5 drops of liquid in the powder capsule Close the capsule and put it 30 s in the triturator (4000–4200 oscillations/min)	12

* Data were obtained from the manufacturers’ Instructions for Use (IFU) and Safety Data Sheets (SDS)

### Adhesive application protocol

All molds were left undisturbed for 15 min to allow the CSCs to set before adhesive application; then the glass slide was removed. Clearfil Tri-S Bond Universal adhesive (Kuraray Noritake Dental Inc., Japan) was applied to the CSC surfaces using two techniques. Each cement group was divided into two subgroups (*n* = 10 each) based on the adhesive application technique: passive or active. In the passive groups, the adhesive was applied with a microbrush, left undisturbed for 5 s, gently air-dried for 5 s, and light-cured for 10 s using a VALO Cordless LED curing unit (Ultradent Products Inc., USA) at 1,200 mW/cm². In the active groups, the adhesive was applied with a microbrush, gently scrubbed for 5 s, then treated as in the passive groups. Only light pressure was applied to minimize disruption of the cement surface.

### Composite build-up

**Fig. 1:**
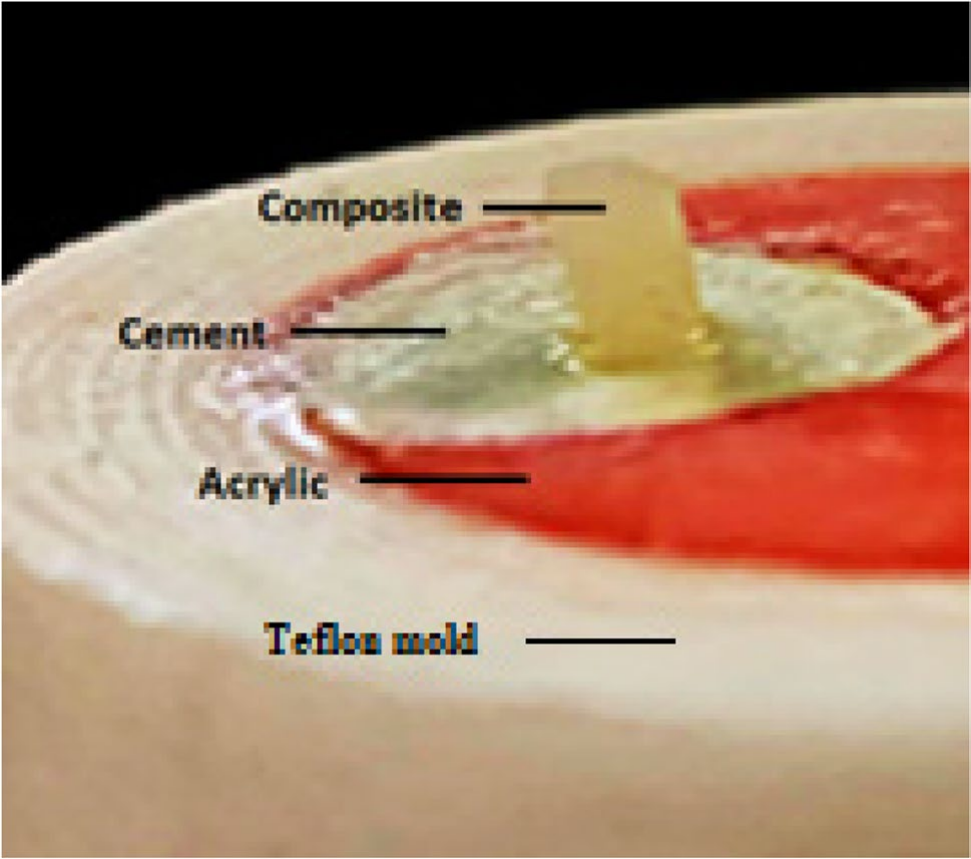
Composite build-up on cement surfaces

A cylindrical starch tube (1.5 mm internal diameter, 2 mm height; Filiz Makarna, Türkiye) was positioned perpendicular to the adhesive-coated surfaces. Clearfil Majesty Flow resin composite (Kuraray Noritake Dental Inc.) was injected into the tubes and cured for 20 s using a VALO Cordless LED curing light at 1200 mW/cm^2^ (Ultradent Products Inc.). All specimens were stored in an incubator at 37 °C and 95% relative humidity for 24 h. The tubes were then carefully removed to expose the composite cylinders attached to the cement surface (Fig. 1).

### Micro-shear bond strength testing

Each specimen was mounted in a universal testing machine (MOD Dental MIC-101, Esetron Smart Robotechnologies, Turkey), and micro-shear bond strength (µSBS) testing was conducted using a chisel-shaped loading device positioned at the cement-composite interface. The load was applied at a crosshead speed of 0.5 mm/min until failure.

The surfaces were examined at 40× magnification using a SOIF Optical Instruments Stereo Microscope (ST6024-B, Turkey). Failure modes were classified into three categories: (A) adhesive failure at the bonding interface; (B) cohesive failure within the composite resin or calcium silicate cement; and (C) mixed failure involving both adhesive and cohesive characteristics. The distribution of failure modes was reported as percentages.

### Sample size calculation

A priori sample size calculation was performed using *G*Power 3.1 (α = 0.05, power = 0.80, two-sided). The expected effect size was derived from published µSBS data on CSC–resin bonding. Çeliksöz and Irmak [16] reported Biodentine–composite µSBS values of 1.8 ± 0.2 MPa at 3 min and 6.0 ± 0.6 MPa at 12 min; based on these values, 10 specimens were included in each subgroup.

### Statistical analysis

The data were analyzed using statistical software (IBM SPSS Statistics V21 Armonk, USA). The normality of data distribution was verified using the Shapiro–Wilk test, and homogeneity of variances was checked with Levene’s test. A one-way analysis of variance (ANOVA) was performed to detect differences among groups. When statistically significant differences were found (*p* < 0.05), post-hoc pairwise comparisons were conducted using Tukey’s test.

## Results

One-way analysis of variance revealed significant differences in SBS among the six groups (*p* < 0.001; Table 2). Tukey’s post-hoc test showed that Biodentine had significantly higher bond strength than White MTA Angelus (AA, AP) and NeoMTA 2 (NA, NP) under both application modes (*p* < 0.05). The lowest mean bond strength was observed in the NeoMTA 2 passive group (NP: 1.0 ± 0.5 MPa), which was significantly (*p* < 0.05) lower than those in both Biodentine subgroups (BA: 4.5 ± 2.8 MPa; BP: 6.1 ± 4.1 MPa). No significant differences were found between passive and active adhesive application modes within the same cement type (Table 2).

**Table 2:** Means and standard deviations of SBS for all experimental groups (MPa)

Group	Mean ± SD
AA	1.49 ± 0.35 ^B^
AP	2.10 ± 0.38 ^B^
NA	1.75 ± 0.72 ^B^
NP	1.02 ± 0.48 ^B^
BA	4.48 ± 2.77 ^A^
BP	6.09 ± 4.09 ^A^

*AA* Angelus MTA Active, *AP* Angelus MTA Passive, *NA* NeoMTA 2 Active, *NP* NeoMTA 2 Passive, *BA* Biodentine Active, *BP* Biodentine Passive

* Homogeneous groups were identified and reported with letter annotations

**Fig. 2:**
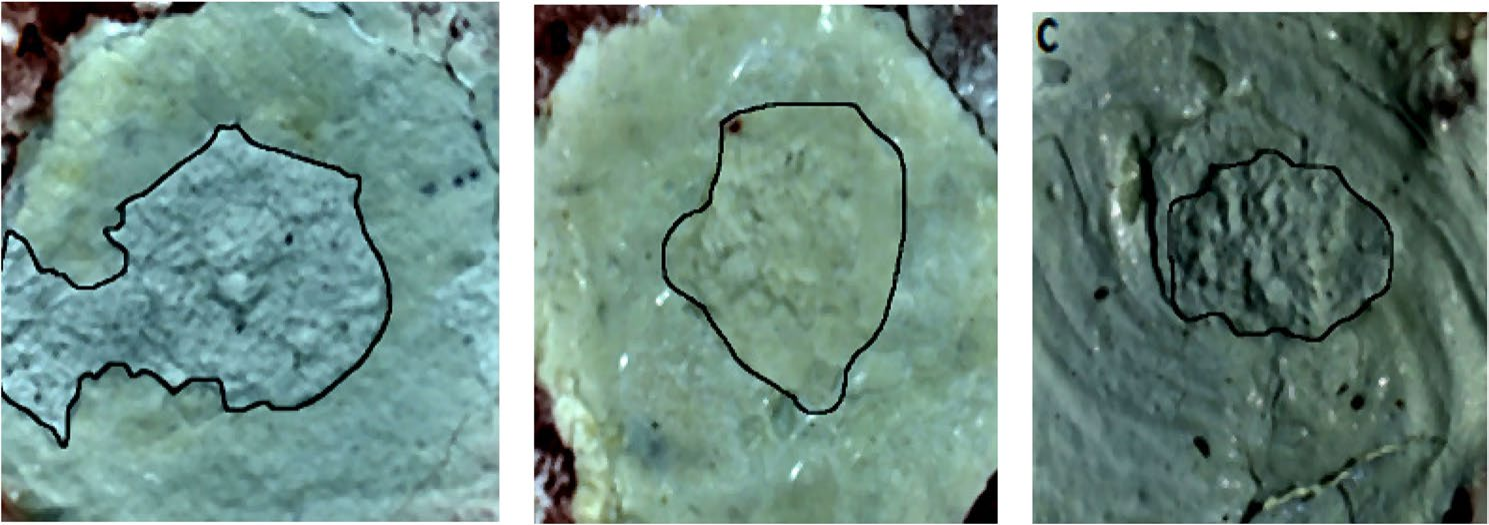
Representative images of cohesive failures within calcium silicate cements (CSCs). **A**–**C** Angelus MTA, Biodentine, and NeoMTA 2: The outlined region indicates the cohesive fracture area within the CSC after debonding. This area may appear non-circular, ? and extend beyond the bonded composite?? due to CSCs weakness and irregular crack propagation within the cement

Failure mode analysis revealed that cohesive failures within the cement predominated in all groups (Table 3). NeoMTA 2 specimens exhibited 100% cohesive failures in both application modes. White Angelus MTA and Biodentine specimens showed predominantly cohesive failures within the cement, along with some mixed failures. No adhesive failures were observed. Representative microscopic images of cohesive failures are presented in Fig. 2.

**Table 3:** Fracture mode of the groups after shear bond strength testing

Group	Cohesive (%)	Adhesive (%)	Mixed (%)
AA	90	–	10
AP	70	–	30
NA	100	–	–
NP	100	–	–
BA	60	–	40
BP	70	–	30

## Discussion

This study evaluated the µSBS of three faster setting CSCs bonded to a flowable resin composite using two adhesive application protocols (passive and active). Bond strength differed significantly among cement brands (*p* < 0.001), whereas the application mode did not significantly affect µSBS within a given cement type. Most failures were cohesive within the cements, indicating that under our test protocol, the measured bond performance was governed primarily by the cement’s intrinsic cohesive strength rather than by adhesion.

Accordingly, the first null hypothesis (no differences among cements) was rejected because Biodentine exhibited significantly higher µSBS than White MTA Angelus and NeoMTA 2, while the second null hypothesis (no difference between passive and active application) was accepted as application mode did not affect µSBS. The third null hypothesis (no difference in fracture mode among materials) was rejected because cohesive failures predominated and were exclusively cohesive for NeoMTA 2.

The significantly higher bond strengths observed for the Biodentine groups is consistent with previous reports indicating that Biodentine can exhibit more favorable early mechanical behavior than MTA Angelus [17, 20, 21]. Biodentine powder is mainly composed of tricalcium silicate (C₃S) ( > ~ 80%), with calcium carbonate and zirconium oxide as additional constituents. Its liquid contains CaCl₂ and a partially modified polycarboxylate; when mixed, these additives are incorporated into the cement, influencing hydration kinetics and the developing microstructure. CaCl₂ accelerates C₃S hydration and increases early strength, while the polycarboxylate adsorbs onto particle surfaces, reduces agglomeration, and improves particle dispersion during setting [2, 13]. MTA Angelus has < 72% tri/dicalcium silicate cement, calcium tungstate and its liquid is water. Differences in cement content accelerators/additives and radiopacifier systems among the cements alter early hydration and microstructure, leading to the differences in µSBS values. Schmidt et al. [17] reported that, depending on the liner/adhesive approach, SBS values measured when bonding was initiated 3–15 min after cement placement ranged from 2.4 to 6.4 MPa for MTA Angelus and from 4.8 to 6.3 MPa for Biodentine; the highest values were obtained when Futurabond NR composite was applied before placing Grandio Flow composite. Notably, bonding was performed immediately after mixing, but the specimens were stored for 28 days under humid conditions before testing. Their larger bonded area, different specimen geometry, and longer cement maturation period may partly explain the higher SBS values compared with our protocol.

The NeoMTA 2 groups (NA, NP) exhibited the lowest bond strength values and demonstrated 100% cohesive failure, indicating that the cement’s early cohesive strength was lower than the adhesive strength at the interface, causing failure within the material under shear loading. Candan et al. [18] reported higher immediate SBS (15 min) for NeoMTA 2 when using a fiber-reinforced bulk-fill composite (4.4 ± 1.7 MPa) compared to our immediate NeoMTA 2 values (NA: 1.8 ± 0.7 MPa). This difference may partly stem from the restorative material: bulk-fill, especially fiber-reinforced formulations, is associated with lower polymerization shrinkage stress, which can reduce pre-test interfacial stresses and early debonding during curing, with substantially higher values observed after delayed restoration (7 days; 10.5 ± 2.4 MPa). This variability supports the concept that, even with an adhesive system, measured bond strength in immediate protocols remains controlled by the cement’s early cohesive/tensile capacity and by differences in polymerization shrinkage stress of the restorative material. Additionally, Falakaloğlu et al. [21] reported mean µSBS values of 15.2 ± 7.4 MPa for Biodentine, 11.5 ± 5.8 MPa for NeoMTA 2, and 6.5 ± 1.9 MPa with a bulk-fill composite resin. In that study, the cements were allowed to set for 24 h before adhesive application, and the restored specimens were stored for an additional 24 h before µSBS testing. Importantly, no adhesive failures were observed in any group. Biodentine showed predominantly mixed failures (80%), with 10% cohesive failure within the bioceramic material. By contrast, NeoMTA 2 and BioMTA+ showed mostly cohesive failures within the bioceramic material (80% and 90%, respectively).

Studies on immediate bonding remain comparatively scarce; however, available reports suggest that using different adhesive strategies and surface pretreatments does not consistently lead to significant improvements in bond strength when cohesive failure within the cement predominates [14, 24, 25]. In contrast, studies in which the cement was allowed to cure for 24 h or more showed that the choice of adhesive protocol and surface pretreatment resulted in significantly different interfacial bond strength values [15, 25–29]. In the present study, the absence of a significant difference in bond strength between passive and active adhesive application within the same cement group suggests that bond strength is determined primarily by the cement’s cohesive integrity rather than by the application mode.

The failure mode findings reinforce this interpretation. Previous investigations frequently report a high incidence of cohesive or mixed fractures in CSCs, typically ranging from approximately 70 to nearly 100% [5, 11, 12, 14, 15, 17, 18, 21, 24, 30]. When cohesive failure predominates, the bond strength values obtained from micro-shear or micro-tensile tests reflect the cement’s early cohesive/tensile capacity at or near the bonded region rather than the true resin–cement interfacial adhesion. Consistent with ISO/TS 11405:2015, bond strength tests are designed to evaluate failure at or near the interface; therefore, predominant cohesive failure indicates that the CSCs were weaker than the adhesive bond at the interface with the flowable resin.

A methodological limitation is that µSBS testing can be suboptimal for CSCs because their relatively low cohesive/tensile strength—particularly at early maturation stages—may increase cohesive failure and thereby confound the interpretation of “interfacial” bond strength. Even after longer maturation periods (7 days), several studies [14, 22, 31, 32] have reported that cohesive failure within the cement often remains the predominant failure mode, indicating that the cement’s intrinsic weakness governed the outcome. Therefore, bond strength results should be complemented with mechanical strength testing (e.g., DTS) and interfacial characterization (e.g., SEM assessment of the adhesive layer and interfacial gap formation, as well as microleakage evaluation) to better contextualize adhesion versus substrate failure.

Clinically, calcium silicate cements have demonstrated favorable outcomes for decades in vital pulp therapy and endodontic repair, where bioactivity and maintenance of a durable coronal seal are the primary determinants of success, rather than strong adhesion between the CSC and the overlying restoration. However, in immediate restoration protocols, the immature mechanical properties of CSC require careful handling and avoidance of excessive pressure during restorative procedures. Accordingly, clinicians should focus on achieving reliable dentin bonding and marginal sealing while selecting CSCs with sufficient early mechanical stability to withstand immediate restoration.

## Conclusion

Within the limits of this in vitro study, Biodentine exhibited significantly higher bond strength values than White MTA Angelus and NeoMTA 2, while NeoMTA 2 showed the lowest bond strengths and exclusively cohesive failures. No significant differences were detected between passive and active adhesive application for any cement type under the tested conditions. The predominance of cohesive failures across groups indicates that at the 24-h time point, the cements’ tensile/cohesive strength was lower than their adhesive bond strength, leading to fractures within the CSC rather than at the interface.

## Data Availability

The datasets generated and analyzed during the current study are available from the corresponding author upon reasonable request.

## Abbreviations

CSC: Calcium Silicate Cement
MPa: MegaPascal
µSBS: Micro-Shear Bond Strength
DTS: Diametral tensile strength

## References

[1] Kaur M, Singh H, Dhillon JS, Batra M, Saini M. MTA versus Biodentine: review of literature with a comparative analysis. J Clin Diagn Res. 2017;11(8):ZG01–5. 28969295 10.7860/JCDR/2017/25840.10374 PMC5620936

[2] Kaup M, Schäfer E, Dammaschke T. An in vitro study of different material properties of Biodentine compared to ProRoot MTA. Head Face Med. 2015;11:16. 25934270 10.1186/s13005-015-0074-9 PMC4424823

[3] Macwan C, Deshpande A. Mineral trioxide aggregate (MTA) in dentistry: a review. J Oral Res Rev. 2014;6(2):71–4.

[4] Walker MP, Diliberto A, Lee C. Effect of setting conditions on mineral trioxide aggregate flexural strength. J Endod. 2006;32:334–6. 16554206 10.1016/j.joen.2005.09.012

[5] Hashem DF, Foxton R, Manoharan A, Watson TF, Banerjee A. The physical characteristics of resin composite–calcium silicate interface as part of a layered/laminate adhesive restoration. Dent Mater. 2014;30(3):343–9. 24418628 10.1016/j.dental.2013.12.010

[6] Septodont USA. Biodentine Instructions for Use. https://www.septodontusa.com/wp-content/uploads/2022/11/Biodentine-IFU.pdf?x92757. Accessed 24 Mar 2026.

[7] Lopez C, Nizami B, Robles A, Gummadi S, Lawson NC. Correlation between dental composite filler percentage and strength, modulus, shrinkage stress, translucency, depth of cure and radiopacity. Mater (Basel). 2024;17(16):3901. 10.3390/ma17163901 PMC11355582 39203079

[8] Al Sunbul H, Silikas N, Watts DC. Polymerization shrinkage kinetics and shrinkage-stress in dental resin composites. Dent Mater. 2016;32(8):998–1006. 27240744 10.1016/j.dental.2016.05.006

[9] Parra Gatica E, Duran Ojeda G, Wendler M. Contemporary flowable bulk-fill resin-based composites: a systematic review. Biomater Investig Dent. 2023;10(1):8–19. 37138762 10.1080/26415275.2023.2175685 PMC10150621

[10] Ranjkesh B, Kopperud HM, Løvschall H. Bond strength of resin-based restorative materials to fast-setting calcium silicate cement using different resin adhesive systems. Eur J Oral Sci. 2024;132(6):e13025. 39462816 10.1111/eos.13025 PMC11602444

[11] Mutluay AT, Mutluay M. Characterisation of the calcium silicate-based cement–composite interface and bonding strength with total-etch or single/two-stage self-etch adhesive systems. Aust Endod J. 2022;48(3):501–9. 34928537 10.1111/aej.12600

[12] Naiboğlu P, Koşar T, Yücel AÇ. Shear bond strength of calcium silicate-based cements to composite resin using a universal adhesive in different application modes: an in vitro study. Aust Dent J. 2024;69(2):102–11. 37875350 10.1111/adj.12990

[13] Pradelle-Plasse N, Mocquot C, Semennikova K, Colon P, Grosgogeat B. Interface between calcium silicate cement and adhesive systems according to adhesive families and cement maturation. Restor Dent Endod. 2020;46(1):e3. 33680892 10.5395/rde.2021.46.e3 PMC7906853

[14] Aksoy S, Ünal M. Shear bond strength of universal adhesive systems to a bioactive dentin substitute (Biodentine) at different time intervals. Stomatol Dis Sci. 2017;1:116–22.

[15] Carretero V, Giner-Tarrida L, Peñate L, Arregui M. Shear bond strength of nanohybrid composite to Biodentine with three different adhesives. Coatings. 2019;9(12):783.

[16] Celiksoz O, Irmak O. Delayed vs. immediate placement of restorative materials over Biodentine and RetroMTA: a micro-shear bond strength study. BMC Oral Health. 2024;24:130. 38273289 10.1186/s12903-024-03917-3 PMC10811922

[17] Schmidt A, Schäfer E, Dammaschke T. Shear bond strength of lining materials to calcium-silicate cements at different time intervals. J Adhes Dent. 2017;19(2):129–35. 28439577 10.3290/j.jad.a38100

[18] Candan M, Altinay Karaca FK, Öznurhan F. Evaluation of the shear bond strength of immediate and delayed restorations of various calcium silicate-based materials with fiber-reinforced composite resin materials. Polym (Basel). 2023;15(19):3971. 10.3390/polym15193971 PMC10575331 37836020

[19] Ergül R, Aksu S, Çalışkan S, Tüloğlu N. Shear bond strength of calcium silicate-based cements to glass ionomers. BMC Oral Health. 2024;24:140. 38281948 10.1186/s12903-024-03890-x PMC10822172

[20] Ipek I, Karaağaç Eskibağlar B, Yildiz S, Ataş O, Ünal M. Analysis of the bond strength between conventional, putty or resin-modified calcium silicate cement and bulk fill composites. Aust Dent J. 2023;68(4):265–72. 37665246 10.1111/adj.12977

[21] Falakaloğlu S, Yeniçeri Özata M, Plotino G. Micro-shear bond strength of different calcium silicate materials to bulk-fill composite. PeerJ. 2023;11:e15183. 37013141 10.7717/peerj.15183 PMC10066686

[22] Palma PJ, Marques JA, Falacho RI, Vinagre A, Santos JM, Ramos JC. Does delayed restoration improve shear bond strength of different restorative protocols to calcium silicate-based cements? Mater (Basel). 2018;11(11):2216. 10.3390/ma11112216 PMC6265959 30413054

[23] Hursh KA, Kirkpatrick TC, Cardon JW, Brewster JA, Black SW, Himel VT, Sabey KA. Shear bond comparison between four bioceramic materials and dual-cure composite resin. J Endod. 2019;45(11):1378–83. 31492579 10.1016/j.joen.2019.07.008

[24] Değer C, Doğan Evcin M, Özduman ZC. Impact of Adhesive Application Modes on Shear Bond Strength of Resin Composites to Biodentine. Eur annals Dent Sci. 2025;52(1):45–9.

[25] Krawczyk-Stuss M, Nowak J, Bołtacz-Rzepkowska E. Bond strength of Biodentine to a resin-based composite at various acid etching times and with different adhesive strategies. Dent Med Probl. 2019;56(1):39–44. 30835973 10.17219/dmp/103589

[26] Sindi AS. An in vitro study to assess the effectiveness of the shear bond strength of mineral trioxide aggregate with different adhesive systems. J Pharm Bioallied Sci. 2021;13(Suppl 1):S672–5. 34447178 10.4103/jpbs.JPBS_689_20 PMC8375844

[27] Bayrak S, Sen Tunç E, Saroğlu I, Eğilmez T. Shear bond strengths of different adhesive systems to white mineral trioxide aggregate. Dent Mater J. 2009;28(1):62–7. 19280969

[28] Shin JH, Jang JH, Park SH, Kim E. Effect of mineral trioxide aggregate surface treatments on morphology and bond strength to composite resin. J Endod. 2014;40(8):1210–6. 25069935 10.1016/j.joen.2014.01.027

[29] Yelamali S, Patil AC. Evaluation of shear bond strength of a composite resin to white mineral trioxide aggregate with three different bonding systems: an in vitro analysis. J Clin Exp Dent. 2016;8(3):e273–7. 27398177 10.4317/jced.52727 PMC4930636

[30] Odabaş ME, Bani M, Tirali RE. Shear bond strengths of different adhesive systems to Biodentine. Sci World J. 2013;2013:626103. 10.1155/2013/626103 PMC3809944 24222742

[31] Alqahtani AS, Sulimany AM, Alayad AS, Alqahtani AS, Bawazir OA. Evaluation of the shear bond strength of four bioceramic materials with different restorative materials and timings. Mater (Basel). 2022;15(13):4668. 10.3390/ma15134668 PMC9267194 35806792

[32] Sulwińska M, Szczesio-Włodarczyk A, Bołtacz-Rzepkowska E. Bond strength of a resin composite to MTA at various time intervals and with different adhesive strategies. Dent Med Probl. 2017;54(2):155–60.

